# Levels of the Novel Endogenous Antagonist of Ghrelin Receptor, Liver-Enriched Antimicrobial Peptide-2, in Patients with Rheumatoid Arthritis

**DOI:** 10.3390/nu12041006

**Published:** 2020-04-06

**Authors:** Vera Francisco, Sulay Tovar, Javier Conde, Jesús Pino, Antonio Mera, Francisca Lago, Miguel Angel González-Gay, Carlos Dieguez, Oreste Gualillo

**Affiliations:** 1 SERGAS (Servizo Galego de Saude) and IDIS (Instituto de Investigación Sanitaria de Santiago), the NEIRID Lab (Neuroendocrine Interactions in Rheumatology and Inflammatory Diseases), Research Laboratory 9, Santiago University Clinical Hospital, 15706 Santiago de Compostela, Spain; vlgfrancisco@gmail.com (V.F.); javier.conde.aranda@sergas.es (J.C.); jesus.pino.minguez@sergas.es (J.P.); 2 Centro de Investigación en Medicina Molecular (CIMUS), Universidade de Santiago de Compostela and Instituto de Investigaciones Sanitarias de Santiago de Compostela (IDIS), 15706 Santiago de Compostela, Spain and CIBER Fisiopatología, de la Obesidad y Nutrición (CIBERobn), Spain; sulay.tovar@usc.es; 3 SERGAS, Santiago University Clinical Hospital, Division of Rheumatology, 15706 Santiago de Compostela, Spain; antonio.mera.varela@sergas.es; 4 Molecular and Cellular Cardiology Group, SERGAS (Servizo Galego de Saude) and IDIS (Instituto de Investigación Sanitaria de Santiago), Research Laboratory 7, Santiago University Clinical Hospital, 15706 Santiago de Compostela, Spain; francisca.lago.paz@sergas.es; 5 Epidemiology, Genetics and Atherosclerosis Research Group on Systemic Inflammatory Diseases, Universidad de Cantabria and IDIVAL, Hospital Universitario Marqués de Valdecilla, 39008 Santander, Spain; miguelaggay@hotmail.com

**Keywords:** ghrelin, growth hormone secretagogue receptor 1a, liver-expressed antimicrobial peptide 2, rheumatoid arthritis

## Abstract

Rheumatoid arthritis (RA) is a debilitating, chronic, inflammatory, autoimmune disease associated with cachexia. The substitutive therapy of gut hormone ghrelin has been pointed at as a potential countermeasure for the management of metabolic and inflammatory complications in RA. The recent discovery of liver-expressed antimicrobial peptide 2 (LEAP2) as an endogenous inverse agonist/antagonist of the ghrelin receptor makes feasible the development of a more rational pharmacological approach. This work aimed to assess the serum LEAP2 levels, in a cohort of RA patients, in comparison with healthy individuals and determine its correlation with inflammatory parameters. LEAP2 levels were determined by a commercial ELISA kit, plasma C-reactive protein (CRP) levels were evaluated using immunoturbidimetry, and serum levels of inflammatory mediators, namely IL-6, IL-8, IL-1β, MIP1α, MCP1, and LCN2, were measured by XMap multiplex assay. LEAP2 serum levels were significantly increased in RA patients (*n* = 101) compared with control subjects (*n* = 26). Furthermore, the LEAP2 levels significantly correlated with CRP and inflammatory cytokines, but not with BMI. These data reveal LEAP2 as a new potential RA biomarker and indicated the pharmacological control of LEAP2 levels as a novel approach for the treatment of diseases with alterations on the ghrelin levels, such as rheumatoid cachexia.

## 1. Introduction

Rheumatoid arthritis (RA) is a debilitating, chronic, inflammatory, autoimmune disease associated with clinical morbidity and mortality, thus having a high socioeconomic impact. It is primarily characterized by the destruction of joint cartilage and bone but is also correlated with an extra-articular manifestation and/or comorbidities, such as rheumatoid cachexia and an increased risk of cardiovascular complications [1]. RA-associated persistent inflammation, combined with reduced physical activity, results in systemic muscle atrophy, prostration, and muscle wasting, whereas dysfunctional fat mass is maintained, which leads to an abnormal metabolic state called rheumatoid cachexia [2]. Since cachexia underlies increased mortality, early and effective therapeutic approaches are urgently required to prevent metabolic dysregulations in RA patients. Accumulating data suggests that inflammation can modulate the secretion and action of gut hormones, which regulate not only appetite, nutrition, energy expenditure, and body mass formation, but also the immune system through interaction with proinflammatory cytokines [3]. Thus, further knowledge of the pathophysiological implications of gut hormones is of relevance to diagnosis and clinical management of metabolic and inflammatory complications in RA.

Ghrelin is primarily a stomach-derived hormone identified as the endogenous ligand of growth hormone secretagogue receptor 1a (GHSR1a). This orexigenic gut hormone stimulates food intake and adiposity, but it has also emerged as a regulator of glucose metabolism, gut motility, reward behaviour, and the immune system, as well as bone and cartilage metabolism, proliferation, and differentiation [4]. RA patients evidenced decreased levels of plasma ghrelin compared with healthy controls [5] and some studies indicated that anti-TNFα therapy transiently augments the levels of this gut hormone [6]. Ghrelin plasma levels are also diminished after induction of inflammation in Freund’s complete adjuvant-induced arthritis in rats and are recovered at day 15, accompanied by a regain of body weight [5]. Osteoblastic cells expressed both GHSR1a and ghrelin, which was pointed to stimulate bone formation through induction of cell proliferation and differentiation, with enhanced mineralization of bone matrix [7]. In cartilage, ghrelin is localized in proliferative and maturation zones and seems to regulate chondrocyte metabolism and biosynthesis of eicosanoids, thus limiting the synthesis of inflammatory prostaglandins and/or leukotrienes [8]. Chondrocytes also expressed ghrelin O-acyltransferase (GOAT), which catalyzed n-octanoyl ghrelin modification, which is necessary for its binding to the GHSR1a [7]. More recently, ghrelin was evidenced to inhibit chondrocytes apoptosis, down-regulate metalloproteinases, and inflammatory cytokines expression and to maintain matrix components expression, leading to the blockage of cartilage degeneration via interaction with GHSR1a [9]. Altogether, these data indicated the protective role of ghrelin in bone and cartilage, thus suggesting the ghrelin system as a potential therapeutic target to RA-associated cachexia and inflammation.

The ghrelin system was considered of high promise in its therapeutic potential in diseases such as obesity, anorexia, or systemic inflammation. These expectations appear only to be fulfilled in cancer cachexia where an administration of ghrelin agonists appears to be of significant clinical benefit [10,11]. Accordingly, liver-expressed antimicrobial peptide 2 (LEAP2), initially known by its role in the innate immune response through disruption of bacterial membrane integrity, was recently identified as the first endogenous GHSR1a ligand with inverse agonist/antagonist properties [12]. LEAP2 blocks the ghrelin-mediated effects, including GHSR1a activation, GH release, food intake, and the maintenance of viable glucose levels during chronic caloric restriction [12]. Moreover, circulating LEAP2 levels are augmented with blood glucose and body mass, and reduced with fasting, in opposition to ghrelin [13]. Therefore, LEAP2 seems to act as a negative feedback regulatory mechanism to ghrelin-induced GHSR1a activation. Knowledge regarding LEAP2 levels in different disease states linked to alterations in the ghrelin system is eagerly awaited since it will allow us to better understand their physiopathology and to improve its targeting, agonist vs antagonist, from a therapeutic point of view. Therefore, we decided to assess the levels of the recently identified endogenous GHSR1a inhibitor LEAP2 in RA patients compared to healthy individuals.

The present study aimed to assess the levels of the recently identified endogenous GHSR1a inhibitor LEAP2 in RA patients compared to healthy individuals. This is the first evidence showing increased LEAP2 serum levels in RA patients, which were correlated with inflammatory mediators, such as C-reactive protein (CRP), interleukin (IL)-6, IL-8, monocyte chemoattractant protein (MCP)-1, and lipocalin (LCN)-2. These results reveal LEAP2 as a potential new biomarker of disease activity and pointed out the LEAP2/ghrelin system as a pharmacological and/or preventive target in rheumatoid arthritis.

## 2. Materials and Methods 

### 2.1. Patients

One hundred and one patients (77 female and 24 male) with an RA diagnosis according to 2010 ACR/EULAR classification criteria (mean age of 54.20 ± 1.449 and BMI of 28.08 ± 0.6249) were enrolled in this study. Of them, 24 patients received no treatment (29.2%) or were being treated with conventional synthetic disease-modifying anti-rheumatic drugs (DMARDs) (45.8%), nonsteroidal anti-inflammatory drugs (NSAIDs) (20.8%), corticosteroids (8.3%), opioids (8.3%), or a combination of these drugs. Fourteen RA-treated patients received anti-TNF therapy (85.7% with Adalimumab and 14.3% with Etanercept). Twenty-six healthy individuals with a mean age of 35.08 ± 2.91 and a BMI of 23.27 ± 0.61 were used as controls. Informed consent was obtained from patients and controls according to the declaration of Helsinki; the study was approved by the Galician Ethical Committee of Clinical Investigation (approved with n° 2014/310). Blood samples were processed and used for human LEAP2 and inflammatory mediators’ determination.

### 2.2. Determination of Serum LEAP2 Levels

Serum LEAP2 levels were determined by a commercial enzyme-linked immunosorbent assay (ELISA) kit (Human LEAP2 [38–77] ELISA kit, Phoenix Pharmaceuticals, Inc) according to the manufacturer’s instructions. Intra-assay and inter-assay variation coefficients were <10% and <15%, respectively. The concentration of circulating LEAP2 (ng/mL) was measured in duplicate for each participant and calculated based on the standard curve generated by the addition of known amounts of human LEAP2. This kit has been previously validated by others [13].

### 2.3. Determination of Serum Inflammatory Mediators

Plasma CRP levels were determined using immunoturbidimetry and measured using a Siemens Advia 2400 analyzer (Siemens AG, Munich, Germany). Serum levels of IL6, IL8, IL1β, MIP1α, MCP1, and LCN2 were evaluated by BioPlex Pro human cytokine (Biorad, Hercules, CA) assay. Data were analyzed with the Bioplex Manager 6.1 software generating a 5-parameter logistic curve fit.

### 2.4. Statistical Analysis

GraphPad Prism 8 (GraphPad Software, San Diego, California, USA) was used for statistical tests. The non-parametric testing between two unpaired groups was performed using the Wilcoxon–Mann–Whitney test. Correlations were determined by Spearman’s correlation coefficient for data which did not follow a normal distribution. Data are expressed as means ± S.E.M, and significance was assumed at *P* < 0.05.

## 3. Results

Our RA patient recruited cohort (Table 1) was non-obese and predominantly female, with a mean age of 54.20 ± 1.449 years. BMI determination indicated a moderate overweight trend (BMI range of 25–29.9) among RA patients compared to a normal weight control population (BMI range of 18.5–24.9), according to the World Health Organization (WHO) classification. Moreover, RA patients demonstrated a statistically significant increase in plasma CRP levels, compared with healthy control individuals. 

**Figure 1:**
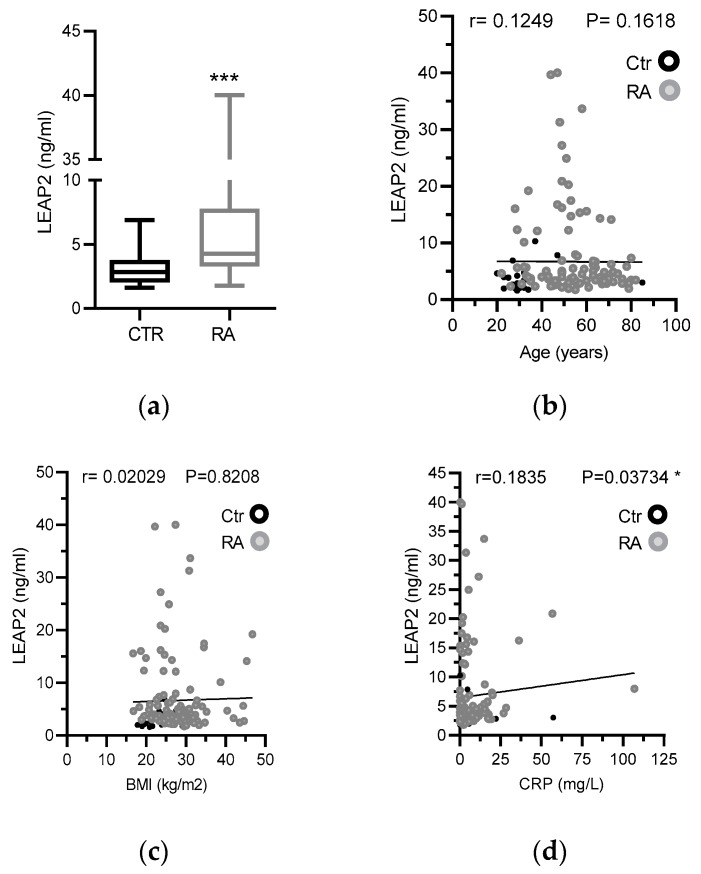
Comparative analysis of Liver-expressed antimicrobial peptide 1 (LEAP2) serum levels in rheumatoid arthritis (RA) patients and healthy controls. (**a**) Plasma levels of LEAP2 protein (ng/mL) were measured by ELISA as described in methods. Data were presented using a vertical box and whiskers graph and compared using the Wilcoxon–Mann–Whitney test. (**b**) Relationship between LEAP2 serum levels and age (years), (**c**) BMI (kg/m^2^) or (**d**) CRP serum levels (mg/L). Data were analyzed by Spearman’s correlation test (r). *** *p* < 0.001.

RA patients also showed a significant increase in LEAP2 serum levels compared with healthy control subjects (Figure 1a). Similar values were found for males and females; thus, gender is declined as a confounding factor (Table 1). Furthermore, a possible interference of age factor was excluded, since there is no correlation between LEAP2 levels and age (Figure 1b). Furthermore, the LEAP2 levels significantly correlated with the inflammatory marker CRP, but not with BMI (Figure 1c,d). These data suggested that LEAP2 levels are associated with an inflammatory status in RA patients, with the irrelevance of BMI.

**Figure 2:**
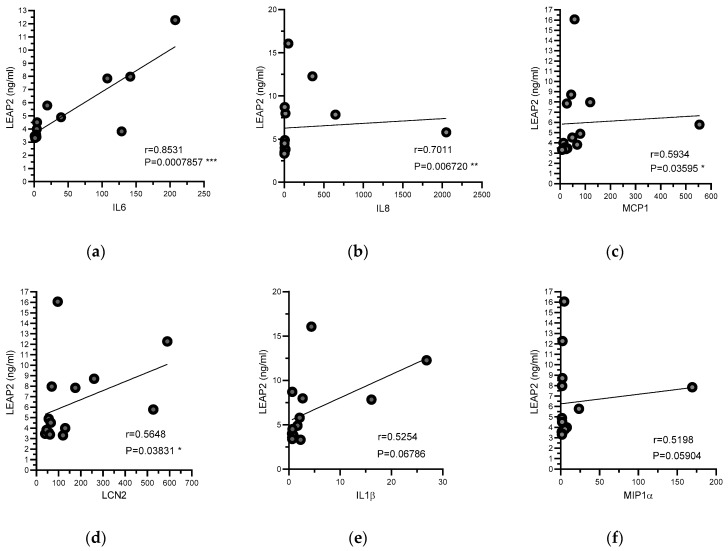
Relationships between levels of serum LEAP2 and proinflammatory mediators. Plasma levels of LEAP2 protein (ng/mL), as well as pro-inflammatory cytokines, were measured in RA-treated patients as described in methods. Relationships between LEAP2 serum levels and (**a**) IL6, (**b**) IL8, (**c**) MCP1, (**d**) LCN2, (**e**) IL1β, and (**f**) MIP1α were determined. Data were analyzed by Spearman’s correlation test (r). * *p* < 0.05, ** *p* < 0.01, *** *p* < 0.001.

In a previously unpublished study, we determined the levels of several inflammatory cytokines and adipokines, such as IL1β, IL6, IL8, MIP1α, MCP1, and LCN2, in 14 RA patients treated with anti-TNFα therapy. In the present study, we analyzed the correlations between LEAP2 levels and these factors in RA patients. In this cohort, LEAP2 levels were positively correlated with IL-6, IL-8, MCP-1, and LCN-2 (Figure 2).

## 4. Discussion

Previous research of our group demonstrated that ghrelin levels are specifically modulated in an experimental model of inflammatory cachexia as well as in patients with RA [5], a degenerative inflammatory autoimmune disease often associated with alterations in body weight homeostasis. In agreement with the results obtained in experimental animals, we demonstrated that RA patients showed ghrelin plasma levels lower than healthy individuals, confirming that ghrelin chronic decrease could be one of the triggers of the RA cachexia. These data, together with others, suggested a potential use of ghrelin substitute therapy as a potential countermeasure for the management of the RA-associated cachexia [6]. The recent discovery of LEAP2 as an endogenous antagonist of the ghrelin receptor makes more feasible the development of a more rational pharmacological approach. This is particularly relevant in the light of new data presented here. Our results showed that LEAP2 levels are higher in RA patients in comparison with healthy individuals. This result, together with previous data showing low ghrelin levels in RA patients, indicate the existence of an inverse relationship between these two peptides. Up to which point, the association of low ghrelin and high LEAP2 levels, meaning an impairment of the ghrelin system, is at the root of or might be a contributing factor to the cachexia and/or the inflammatory state in RA remains to be established. Our results could also be in keeping with the hypothesis that LEAP2 might inhibit the production or secretion of ghrelin. Although there is still no clear evidence for this, in acute phase responses from bacterial infections of H. pylori, LEAP2 expression is elevated [14]. In line with this, ghrelin levels are low in H. pylori infections whereas eradication of the infection restored ghrelin levels [15]. To note, according to our results, LEAP2 levels correlated positively with CRP levels, suggesting that LEAP2 is strongly influenced by acute phase response factors. In this regard, in RA patients treated with anti-TNF therapy, LEAP2 levels correlated positively with other inflammatory parameters, such as IL6, IL-8, MCP-1, and LCN2, suggesting that inflammatory status is crucial to the modulation of LEAP2.

The limitations of this study include an unbalanced number of subjects in the control and RA populations due to the challenges in recruiting control individuals. Furthermore, it is observed that there is a moderate overweight trend in RA patients, with a mean age higher than control subjects. BMI is widely used for determining healthful weight, but it is unable to distinguish between lean and fat mass. So, although BMI values indicated an overweight RA population, these individuals could demonstrate RA-associated cachexia, characterized by systemic muscle atrophy and wasting, whereas dysfunctional fat mass and high levels of inflammatory cytokines are maintained. Besides BMI, determination of waist circumference, body mass index, body fat percentage, and other measures of adiposity will be of interest in future research on the role of LEAP2 in RA pathology. Here, we revealed that LEAP2 serum levels were positively correlated with inflammatory cytokines and adipokines in patients under anti-TNF-α therapy. However, we cannot rule out the impact of different therapeutic approaches on LEAP2 serum levels in RA patients.

In conclusion, this is the first evidence showing that, in RA patients, LEAP2 levels are higher than in healthy individuals uncovering a new potential biomarker of disease activity. Our results suggest that pharmacological control of LEAP2 levels might be effective for the treatment of diseases with alterations of ghrelin levels, such as rheumatoid cachexia. Therefore, future research is needed to evaluate the implications of these data to the clinical practice.

## Figures and Tables

**Table 1:** Anthropometric and laboratory characteristics of the studied groups.

Parameters	Control Population (*n* = 26; 13 female, 13 male)	Rheumatoid Arthritis (RA) Patients (*n* = 101; 77 female, 24 male)
Age (years)	*male* 32.77 ± 2.595 *female* 37.38 ± 5.25 *total* 35.08 ± 2.906	*male* 55.33 ± 2.907 *female 53.84* ± 1.679 *total* 54.20 ± 1.449 ***
BMI (Kg/m^2^)	*male* 25.34 ± 0.7835 *female* 21.2 ± 0.49.39 *total* 23.27 ± 0.6142	*male* 27.98 ± 1.106 *female 28.11* ± 0.7456 *total* 28.08 ± 0.6249 ***
CRP (mg/L)	*male* 1.56 ± 0.7076 *female* 6.715 ± 4.529 *total* 4.138 ± 2.304	*male* 9.519 ± 4.382 *female 7.162* ± 1.093 *total* 7.722 ± 1.324 ***
Liver enriched antimicrobial peptide 2 (LEAP2) (ng/mL)	*male* 3.356 ± 0.4130 *female 2.738* ± 0.2257 *total* 3.047 ± 0.2387	*male* 7.395 ± 1.692 *female 7.751* ± 0.8959 *total* 7.666 ± 0.7885 ***

*** *p* < 0.001.
